# Play

**Published:** 1888-06-09

**Authors:** 


### PLAY.

"What is play?" asks Dr. Strachan, in a book published some years ago; and he answers his own question thus: "Play may be defined as all voluntary exercise in the young, prompted by inclination and producing pleasure." He points out that this instinct for play is divinely implanted in order "to secure in the young the exercise required for development, just as the appetite for food is intended to secure proper nourishment." In this aspect of play consider the position of our city children. They are impelled by a restless, ceaseless instinct, and not by the Devil, as the landlords and the police seem to think. Pent up in narrow streets and in back courts, without a bit of space which they can call their own, their play inevitably becomes in great part mischief. What can a poor boy do but pull bricks out of the walls of the ashpit to build houses with, or climb upon its roof and tear the slates off to make traps for the city sparrows? If they fly kites the policeman cuts the string; if they dig holes in the court to play at marbles, some one denounces them to the police; if they play ball against the wall, the policeman grabs the ball; if they make slides on the pavement, he puts salt upon them; if they try to swim in the river, they are almost poisoned by the sewage, and when they come out it is to find the man in blue waiting for them beside their clothes; if they pitch a wicket on an empty building site, the sound of the well-known whistle stops the game before they have completed their innings.

